# Medical Jurisprudence

**Published:** 1862-01

**Authors:** 


MEDICAL JURISPRUDENCE.—By ALFRED SWAYNE TAYLOR, M. D., F. R. S., Fellow of the Royal College of Physicians; Hon. M. D. Univ. St. Andrews; Member of the Royal College of Surgeons; and Professor of Medical Jurisprudence and Chemistry in Guy's Hospital. *Qui nesci ignorare ignorat scire.* Fifth American, from the Seventh and Revised London Edition. Edited, with Additions, by EDWARD HARTSHORNE, M.D., one of the Surgeons to the Pennsylvania Hospital. Philadelphia: BLANCHARD & LEA. 1861.

To those, already familiar with the former editions of Dr. TAYLOR's work, it will be only necessary to quote from the author's preface to the sixth London edition, to show how radically the present volume has been improved and amended: During the last fourteen years, improvements in medicine and jurisprudence have taken place to so great an extent that a practitioner, whether of the medical or legal profession, would, in the earlier editions, meet with deficiencies which on the present occasion it has been his special object to supply.

In the section on POISONS, a modification of the definition of the term poison, and of the act of poisoning, has been rendered necessary by crimes of recent date. Additions have been made on poisoning by ammonia,—chronic poisoning by arsenic,—the absorption and detection of arsenic in the dead body,—poisoning by arseniuretted hydrogen,—the detection of absorbed mercury,—poisoning by Scheele's green, tartar emetic, locust beans, prussic acid, nux vomica, and strychnia. The chapter on the two last-named poisons has been entirely re-written. The reader will also find additional facts and cases in the sections on poisoning by œnanthe crocata, aconite, and lobelia. The details regarding poisons are, however, given throughout in a concise form, as constituting only a part of the general science of Medical Jurisprudence. A new edition of Dr. Taylor's work on Poisons has been published by Blanchard & Lea, and to this the reader is referred for that special information on facts, whether of a legal, medical, or medico-legal kind, which belong to the subject of Toxicology.

In the section on Wounds, the additions include the rules respecting dying declarations made to medical men,—the detection of blood on weapons and clothing,—the medico-legal examination of wounds,—the microscopical and chemical analysis of blood,—cicatrices,—locomotion after severe injuries,—and the effects of concussion of the brain and spinal marrow, illustrated by recent cases.

Additional facts have been introduced into the sections on Pregnancy, Delivery and Abortion; and new cases are appended to the subjects of Tenancy by Courtesy, Protracted Gestation, and Legitimacy. In the chapter on Rape, additions have been made to the medical proofs of rape on infants and children.

The chapters on Drowning and other forms of death by Asphyxia will be found to contain new facts and cases; and, lastly, in the chapter on Insanity, the sections on Homicidal Mania and Dipsomonia have been corrected and enlarged.

